# Prognostic and predictive value of CD163 expression and the CD163/CD68 expression ratio for response to adjuvant chemotherapy in patients with surgically resected lung squamous cell carcinoma

**DOI:** 10.1111/1759-7714.14937

**Published:** 2023-05-20

**Authors:** Naoki Yanagawa, Shunsuke Shikanai, Mayu Sugai, Yoshihiko Koike, Yoshinari Asai, Takayuki Tanji, Ryo Sugimoto, Mitsumasa Osakabe, Noriyuki Uesugi, Hajime Saito, Makoto Maemondo, Tamotsu Sugai

**Affiliations:** ^1^ Department of Molecular Diagnostic Pathology Iwate Medical University Yahaba‐cho Japan; ^2^ Department of Thoracic Surgery Iwate Medical University Yahaba‐cho Japan; ^3^ Division of Pulmonary Medicine, Department of Internal Medicine Iwate Medical University Yahaba‐cho Japan

**Keywords:** chemotherapy, lung squamous cell carcinoma, prognosis, tumor‐associated macrophage (TAM)

## Abstract

**Background:**

Macrophages infiltrating the tumor microenvironment are defined as tumor‐associated macrophages (TAMs). TAMs can be polarized into different phenotypes, that is, proinflammatory M1 macrophages or anti‐inflammatory M2 macrophages. Particularly, M2 macrophages promote angiogenesis, wound healing, and tumor growth. This study aimed to evaluate whether M2 TAMs can serve as a useful marker to predict prognosis and benefit from adjuvant chemotherapy in patients with surgically resected lung squamous cell carcinomas (SCCs).

**Methods:**

We examined 104 patients with SCC. Tissue microarrays were constructed, and the density of TAMs was analyzed by immunohistochemistry for expression of CD68 and CD163. The relationship between CD68 and CD163 expression and the CD163/CD68 expression rate and clinicopathological characteristics including patient outcomes were investigated. In addition, propensity score matching (PSM) analysis was conducted to test the hypothesis that these cells significantly influenced chemotherapy responses.

**Results:**

Univariate analysis revealed that pathological stage, CD163 expression, and the CD163/CD68 expression ratio were significant prognostic factors. Multivariate analysis showed that these factors were all independent prognostic factors. Thirty‐four pairs were determined by using PSM analysis. Patients with a low CD163/CD68 expression ratio benefited more from adjuvant chemotherapy than those with a high ratio.

**Conclusion:**

We suggest that M2 TAMs may be a useful marker to predict prognosis and differential benefit from adjuvant chemotherapy in patients with surgically resected lung SCCs.

## Graphical abstract

CD163 expression and the CD163/CD68 expression ratio were associated with patient survival and these were independent predictive factors of patient survival. Patients with a low CD163/CD68 expression ratio benefited more from adjuvant chemotherapy than those with a high CD163/CD68 expression ratio.

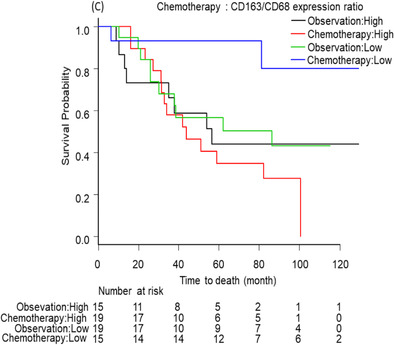

## INTRODUCTION

Lung cancer (LC) is the leading cause of cancer‐related death in developed countries.^1^ Lung squamous cell carcinoma (SCC) is the second most common subtype of LC following lung adenocarcinoma (ADC).^2^ However, unlike ADC, most SCCs do not contain driver mutations and respond less favorably to targeted therapy.^3^ Recently, immune checkpoint inhibitors (ICIs), such as pembrolizumab, have been demonstrated to exhibit superior clinical efficacy against various types of cancer, including non‐small cell lung cancer (NSCLC).^4^, ^5^ However, these ICIs have been reported to be less effective in patients with programmed death‐ligand 1 (PD‐L1)‐negative tumors.^6^, ^7^ Therefore, it is necessary and important to predict the benefit of adjuvant chemotherapy in SCCs without these mutations in target genes or PD‐L1 expression.

Cancer cells interact with surrounding stromal cells via complex mechanisms, making up the tumor microenvironment (TME).^8^ Within the last decade, the TME has been shown to be important for the proliferation, invasion, metastasis, and chemoresistance of cancer cells.^9^, ^10^ Macrophages infiltrating the TME are defined as tumor‐associated macrophages (TAMs),^11^ and can be polarized into different phenotypes, that is, proinflammatory M1 macrophages (classical type) or anti‐inflammatory M2 macrophages (alternative type).^12^, ^13^ Particularly, M2 TAMs promote angiogenesis, wound healing, and tumor growth.^12^, ^13^ Previous clinical studies in NSCLC demonstrated that high infiltration of M1 TAMs into tumor islets was associated with increased survival,^14^, ^15^ whereas a high infiltration of M2 TAMs into tumor islets and tumor stroma was associated with reduced survival.^7^, ^14^, ^16^ Therefore, the presence of M2 TAMs may be considered a prognostic marker; however, to our knowledge, no reports have assessed M2 TAMs as a predictive marker for chemotherapy in lung SCC.

This study aimed to evaluate whether TAMs are a useful marker to predict prognosis and benefit from adjuvant chemotherapy in patients with surgically resected lung SCCs.

## METHODS

### Patients

A retrospective review of a prospectively maintained surgical database was performed to identify patients who underwent primary LC resection with curative intent from 2010 to 2016 at Iwate Medical University Hospital. The histopathological diagnosis was made according to the eighth edition of the TNM Classification of the Union for International Cancer Control and the 2021 World Health Organization classification.^2^, ^17^ Patients were excluded from the current evaluation if they underwent chemotherapy or radiotherapy before surgery, underwent incomplete resection, had multiple primary lung cancers, or had incomplete follow‐up data. Finally, 104 patients with SCC were examined. Patient survival was confirmed through the medical record and telephone interviews. The end of the follow‐up period was May 2021 (median follow‐up period: 58.2 months; maximum: 135.3 months; minimum: 4.9 months). This study was approved by the Institutional Review Board of Iwate Medical University (approval no. MH2021‐068) and was conducted according to the principles of the Declaration of Helsinki. Written informed consent was waived because this was a retrospective study, the patient data remained anonymous, and an opt‐out approach was used.

### Preparation of tissue samples and tissue microarrays (TMAs)

Paraffin‐embedded tissues used for the construction of TMAs were stored at room temperature. These materials showed sufficient quality for biological assessments, including high DNA and RNA quality. TMAs were created using a manual tissue array (Azumaya Co., Tokyo, Japan). A representative area with both marked infiltrated immune cells in the peritumoral stroma and tumor cells, as assessed by hematoxylin and eosin (H&E) stain of the whole tissue section because of avoiding effect of heterogeneity, was selected for inclusion in the TMA. Tissue cores (5 mm) were collected from target lesions and placed into recipient blocks containing 12 cores, including 10 cancer tissues and two control tissues. After construction, 3 μm‐thick sections were prepared and stained with H&E using the initial slides to verify the histological diagnosis. Finally, serial sections were cut from the TMA blocks for immunohistochemical staining.

### Immunohistochemistry

TMA blocks were sliced into 4 μm‐thick sections, deparaffinized, and stained for CD68 (clone PG‐M1; Dako) as a pan macrophage marker and CD163 (clone 10D6; Leica Biosystems) as an M2 macrophage marker using a DAKO Autostainer Universal Staining System (Dako). In addition, TMAs were stained for PD‐L1 using PD‐L1 IHC 22C3 pharmDx assays (Agilent Technologies, Inc.) on an Autostainer Link 48 using an automated staining protocol.

### Immunohistochemical evaluation

**FIGURE 1:**
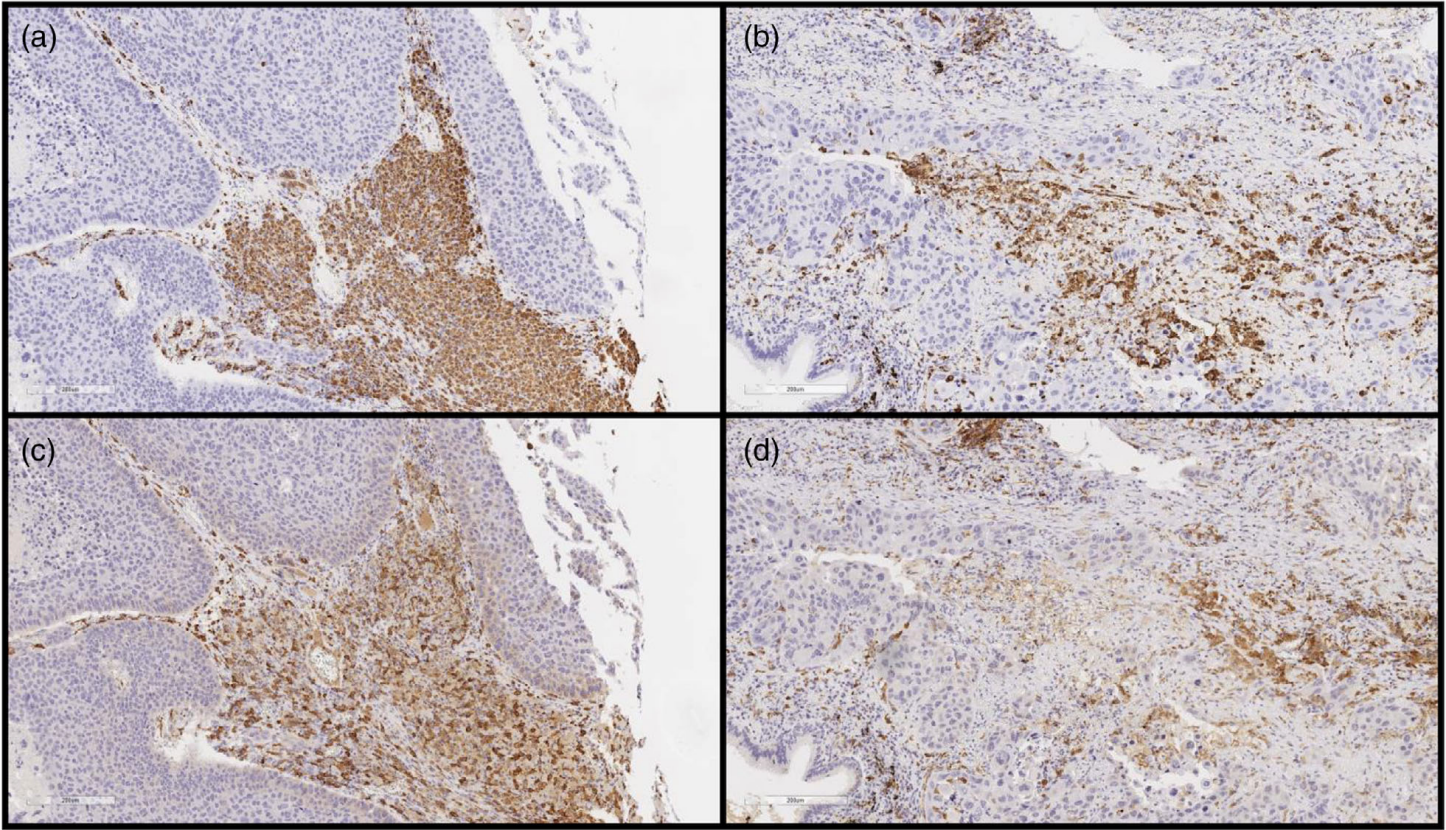
Representative images of immunohistochemistry. (a) and (b) CD68 staining. (c) and (d) CD163 staining.

The expression of CD68 or CD163 was defined as a granular cytoplasm or a cytoplastic and membrane staining pattern. Representative images are shown in Figure 1. The number of positive cells in each high‐power field (HPF; 400x magnification) was calculated by two independent pathologists (S.S. and N.Y.) in a blind manner. The stained cells in 10 consecutive HPFs in hot spot areas were calculated to analyze macrophage infiltration. The final TAM density for each case equated to an average of the results obtained by the two examiners and expressed as cell number/10 HPFs, as described previously.^11^ For PD‐L1, sections showing greater than 1% PD‐L1 immunohistochemical expression in the tumor cells (TCs) and surrounding immune cells (ICs) were considered positive.

### Statistical analysis

Statistical comparisons were performed using *χ* ^2^ or Fisher's exact tests, as appropriate. Recurrence‐free survival (RFS) and overall survival (OS) were analyzed using the Kaplan–Meier method, and differences in variables were calculated using log‐rank tests. RFS was defined as the time from surgery to recurrence, death, or the last follow‐up. OS was defined as the time from surgery to death of any cause or the last follow‐up. The last follow‐up observation was censored if the patient was alive or lost to follow‐up. Multivariate survival analysis was performed using the Cox proportional hazards model. To test the hypothesis that chemotherapy significantly affected outcomes, propensity score matching (PSM) analysis was conducted with regard to chemotherapy and observation of CD68, CD163 expression, and the ratio of CD163 expression/CD68 expression, to assess treatment effects. A logistic regression model including the following covariates was used to estimate the propensity score: age, sex, smoking status, tumor stage, pleural invasion, lymphocytic invasion, and vascular invasion. We performed 1:1 matching using the propensity score with a caliper width of ≤0.2 standard deviations (SD). All statistical analyses were performed using EZR (Saitama Medical Center),^18^ which is a modified version of R commander (The R Foundation for Statistical Computing) designed to add functions frequently used in biostatistics 18 or using JMP Pro 16.1 software (SAS). Results with *p*‐values < 0.05 were considered statistically significant.

## RESULTS

### Patient characteristics and treatment

The patient characteristics and treatments are summarized in Table 1. The tumors from 97 males (93.3%) and seven females (6.7%) with an average age of 71.2 (range, 51–87) years were examined. A total of 102 patients were smokers, and 57 tumors (54.8%) were classified as stage I, 32 (30.8%) as stage II, and 15 (14.4%) as stage III. Pleural invasion was detected in 28 patients (26.9%), lymphocytic invasion in 12 patients (11.5%), and vascular invasion in 34 patients (32.7%). After surgery, 33 patients received tegafur uracil, whereas 29 received platinum‐doublet chemotherapy, including carboplatin plus gemcitabine, tegafur/gimeracil/oteracil, or paclitaxel, as adjuvant chemotherapy. In total, 62 (59.6%) out of 104 patients received adjuvant chemotherapy.

**TABLE 1:** Patient characteristics and treatment.

Characteristics	No. (%)
Sex	
Female	7 (6.7)
Male	97 (93.3)
Age (average, years)	51–87 (71.2)
Smoking	
No	2 (1.9)
Yes	102 (98.1)
Stage	
I	57 (54.8)
II	32 (30.8)
III	15 (14.4)
Pleural invasion	
No	76 (73.1)
Yes	28 (26.9)
Lymphatic invasion	
No	92 (88.5)
Yes	12 (11.5)
Vascular invasion	
No	70 (67.3)
Yes	34 (32.7)
Adjuvant chemotherapy	
No (observation)	42 (40.4)
Yes (chemotherapy)	62 (59.6)

### Infiltration of cells expressing CD68 and CD163, and PD‐L1 expression in the TME

Expression of CD68, CD163, the CD163/CD68 ratio, and PD‐L1 are summarized in Table 2. Briefly, the range of CD68+ cell counts/10HPFs was 130–1260 (median: 643). Based on this, we grouped tumors as low expression when they had <643 cells, and high expression when the cell counts were above 643. Similarly, the range of CD163+ cell counts/10HPFs was 86–660 (median: 346), and groups were split into high and low‐expression groups based on the median of 346. The range of the CD163/CD68 count ratio was 0.31–0.9 (median: 0.52). Cases were also split into high and low CD163/CD68 count ratios based on the median of 0.52. Group distribution is depicted in Table 2B. Tumoral expression of PD‐L1 (TC) was detected in 44 (42.3%) of 104 cases, and immune cell PD‐L1 expression (IC) was detected in 41 (39.4%) of 104 cases (Table 2C).

**TABLE 2:** CD68, CD163, and PD‐L1 expression.

(A)	Counts or ratio
CD68 counts/10HPFs (median)	130–1260 (643)
CD163 counts/10HPFs (median)	86–660 (346)
CD163/CD68 count ratio (median)	0.31–0.9 (0.52)

(B)	No. (%)
Low CD68 expression	52 (50%)
High CD68 expression	52 (50%)
Low CD163 expression	52 (50%)
High CD163 expression	52 (50%)
Low CD163/CD68 expression ratio	52 (50%)
High CD163/CD68 expression ratio	52 (50%)

(C)	No. (%)
PD‐L1 (TC)	
Negative expression	60 (57.7%)
Positive expression	44 (42.3%)
PD‐L1 (IC)	
Negative expression	63 (60.6%)
Positive expression	41 (39.4%)

Abbreviations: HPF, high power field; IC, immune cell; PD‐L1, programmed cell death ligand 1; TC, tumor cell.

### Relationship between expression of CD68, CD163, and the CD163/CD68 ratio and clinicopathological characteristics

The associations between clinicopathological characteristics and CD68 and CD163 expression and the ratio of CD163/CD68 expression are shown in Table 3. High CD163 expression was more frequently found in patients with tumoral PD‐L1 expression (TC) (*p* = 0.029).

**TABLE 3:** The relationship between the expression of CD68 and CD163 and the CD163/CD68 ratio with clinicopathological characteristics.

Variables	CD68 expression				CD163 expression			CD163/CD68 count ratio	
	Low (52, %)	High (52, %)	*p*‐value	Low (52, %)	High (52, %)	*p*‐value	Low (52, %)	High (52, %)	*p*‐value
Sex									
Female (7)	4 (57.1)	3 (42.9)	1	5 (71.4)	2 (28.6)	0.437	5 (71.4)	2 (28.6)	0.437
Male (107)	48 (49.5)	49 (50.5)		47 (48.5)	50 (51.5)		47 (48.5)	50 (51.5)	
Age (average)	71.3	71.1	0.931	70.9	71.4	0.756	70.9	71.5	0.719
Smoking									
No (2)	2 (100)	0	0.495	2 (100)	0	0.495	1 (50)	1 (50)	1
Yes (102)	50 (49)	52 (51)		50 (49)	52 (51)		51 (50)	51 (50)	
Stage									
I (57)	29 (50.9)	28 (49.1)	0.572	28 (49.1)	29 (50.9)	0.688	29 (50.9)	28 (49.1)	0.893
II (32)	14 (43.8)	18 (56.2)		15 (46.9)	17 (53.1)		15 (46.9)	17 (53.1)	
III (15)	9 (60)	6 (40)		9 (60)	6 (40)		8 (53.3)	7 (46.7)	
Pleural invasion									
No (76)	39 (51.3)	37 (48.7)	0.825	39 (51.3)	37 (48.7)	0.825	39 (51.3)	37 (48.7)	0.825
Yes (28)	13 (46.4)	15 (53.6)		13 (46.4)	15 (53.6)		13 (46.4)	15 (53.6)	
Lymphatic invasion									
No (92)	44 (47.8)	48 (52.2)	0.358	46 (50)	46 (50)	1	49 (53.3)	43 (46.7)	0.122
Yes (12)	8 (66.7)	4 (33.3)		6 (50)	6 (50)		3 (25)	9 (75)	
Vascular invasion									
No (70)	38 (54.3)	32 (45.7)	0.296	39 (55.7)	31 (44.3)	0.143	40 (57.1)	30 (42.9)	0.059
Yes (34)	14 (41.2)	20 (58.8)		13 (38.2)	21 (61.7)		12 (35.3)	22 (64.7)	
PD‐L1 (TC)									
Negative expression (60)	31 (51.7)	29 (48.3)	0.843	36 (60)	24 (40)	0.029	33 (55)	27 (45)	0.321
Positive expression (44)	21 (47.7)	23 (52.3)		16 (36.4)	28 (63.6)		19 (43.2)	25 (56.8)	
PD‐L1 (IC)									
Negative expression (62)	35 (56.5)	27 (43.5)	0.162	34 (54.8)	28 (45.2)	0.318	33 (53.2)	29 (46.8)	0.549
Positive expression (42)	17 (40.5)	25 (59.5)		18 (42.9)	24 (57.1)		19 (45.2)	23 (54.8)	

Abbreviations: IC, immune cell; PD‐L1: programmed cell death ligand 1; TC, tumor cell.

### Univariate and multivariate analyses of overall survival and recurrence‐free survival

The mean follow‐up period was 58.2 months (range, 4.9–135.3 months); 59 of the 104 patients died during the follow‐up period. Of these, 39 died due to cancer recurrence, and the remaining 20 died of other causes. Of the 45 patients alive at the time of analysis, six had recurrent disease, while 39 had no evidence of disease.

The 5‐year OS rate was 52.5%. Univariate analysis revealed that pathological stage, CD163 expression, and the CD163/CD68 expression ratio were significant prognostic factors for OS (Table 4A,B). Multivariate analysis showed that the pathological stage, CD163 expression (OS; hazard ratio [HR] = 2.85, 95% confidence interval [CI]: 1.65–3.41, *p* < 0.001), and the CD163/CD68 expression ratio (OS; HR = 2.37, 95% CI: 1.38–4.05, *p* = 0.002) were independent prognostic factors for OS.

**TABLE 4:** Univariate and multivariate analyses based on overall survival (A and B) and recurrence‐free survival (C and D).

(A)				
Factor	Univariate analysis		Multivariate analysis	
	HR (95% CI)	*p*‐value	HR (95% CI)	*p*‐value
Sex (M vs. F)	1.5 (0.46–4.95)	0.503		
Age	1.01 (0.98–1.05)	0.459		
p‐stage (II/III vs. I)	1.77 (1.06–2.97)	0.03	2.02 (1.2–3.41)	0.008
Pleural invasion (positive vs. negative)	1.03 (0.58–1.81)	0.933		
Lymphatic invasion (positive vs. negative)	1.53 (0.77–3.05)	0.228		
Vascular invasion (positive vs. negative)	1.65 (0.96–2.83)	0.071		
CD68 count median (high vs. low)	1.57 (0.93–2.65)	0.092		
CD168 count median (high vs. low)	2.6 (1.52–4.47)	<0.001	2.85 (1.65–3.41)	<0.001

(B)				
Factor	Univariate analysis		Multivariate analysis	
	HR (95%CI)	*p*‐value	HR (95%CI)	*p‐*value
Sex (M vs. F)	1.5 (0.46–4.95)	0.503		
Age	1.01 (0.98–1.05)	0.459		
p‐stage (II/III vs. I)	1.77 (1.06–2.97)	0.03	1.85 (1.1–3.11)	0.019
Pleural invasion (positive vs. negative)	1.03 (0.58–1.81)	0.933		
Lymphatic invasion (positive vs. negative)	1.53 (0.77–3.05)	0.228		
Vascular invasion (positive vs. negative)	1.65 (0.96–2.83)	0.071		
CD163/CD68 count ratio (high vs. low)	2.24 (1.31–3.82)	0.003	2.37 (1.38–4.05)	0.002

(C)				
Factor	Univariate analysis		Multivariate analysis	
	HR (95% CI)	*p*‐value	HR (95% CI)	*p*‐value
Sex (M vs. F)	1.36 (0.48–3.83)	0.565		
Age	1.01 (0.98–1.04)	0.564		
p‐stage (II/III vs. I)	1.81 (1.11–2.96)	0.018	2.06 (1.23–3.43)	0.006
Pleural invasion (positive vs. negative)	1.17 (0.69–1.99)	0.567		
Lymphatic invasion (positive vs. negative)	1.55 (0.8–2.99)	0.193		
Vascular invasion (positive vs. negative)	1.74 (1.04–2.94)	0.037	1.24 (0.71–2.16)	0.446
CD68 count median (high vs. low)	1.35 (0.82–2.21)	0.24		
CD168 count median (high vs. low)	2.07 (1.26–3.4)	0.004	2.24 (1.31–3.83)	0.003

(D)				
Factor	Univariate analysis		Multivariate analysis	
	HR (95% CI)	*p*‐value	HR (95% CI)	*p*‐value
Sex (M vs. F)	1.36 (0.48–3.83)	0.565		
Age	1.01 (0.98–1.04)	0.564		
p‐stage (II/III vs. I)	1.81 (1.11–2.96)	0.018	1.86 (1.13–3.07)	0.015
Pleural invasion (positive vs. negative)	1.17 (0.69–1.99)	0.567		
Lymphatic invasion (positive vs. negative)	1.55 (0.8–2.99)	0.193		
Vascular invasion (positive vs. negative)	1.74 (1.04–2.94)	0.037	1.35 (0.79–2.33)	0.274
CD163/CD68 count ratio (high vs. low)	2.13 (1.29–3.53)	0.003	2.09 (1.24–3.55)	0.006

Abbreviations: CI, confidence interval; F, female; HR, hazard ratio; M, male.

The 5‐year RFS rate was 46%, and univariate analysis revealed that pathological stage, vascular invasion, CD163 expression, and the CD163/CD68 expression ratio were significant predictive factors for recurrence (Table 4C,D). Multivariate analysis showed that pathological stage, CD163 expression (RFS; HR = 2.24, 95% CI: 1.31–3.83, *p* = 0.003), and the CD163/CD68 expression ratio (RFS; HR = 2.09, 95% CI: 1.24–3.55, *p* = 0.006) were independent predictive factors for recurrence.

### Survival outcome in months according to the expression status of CD68 and CD163 and treatment after propensity score matching

**FIGURE 2:**
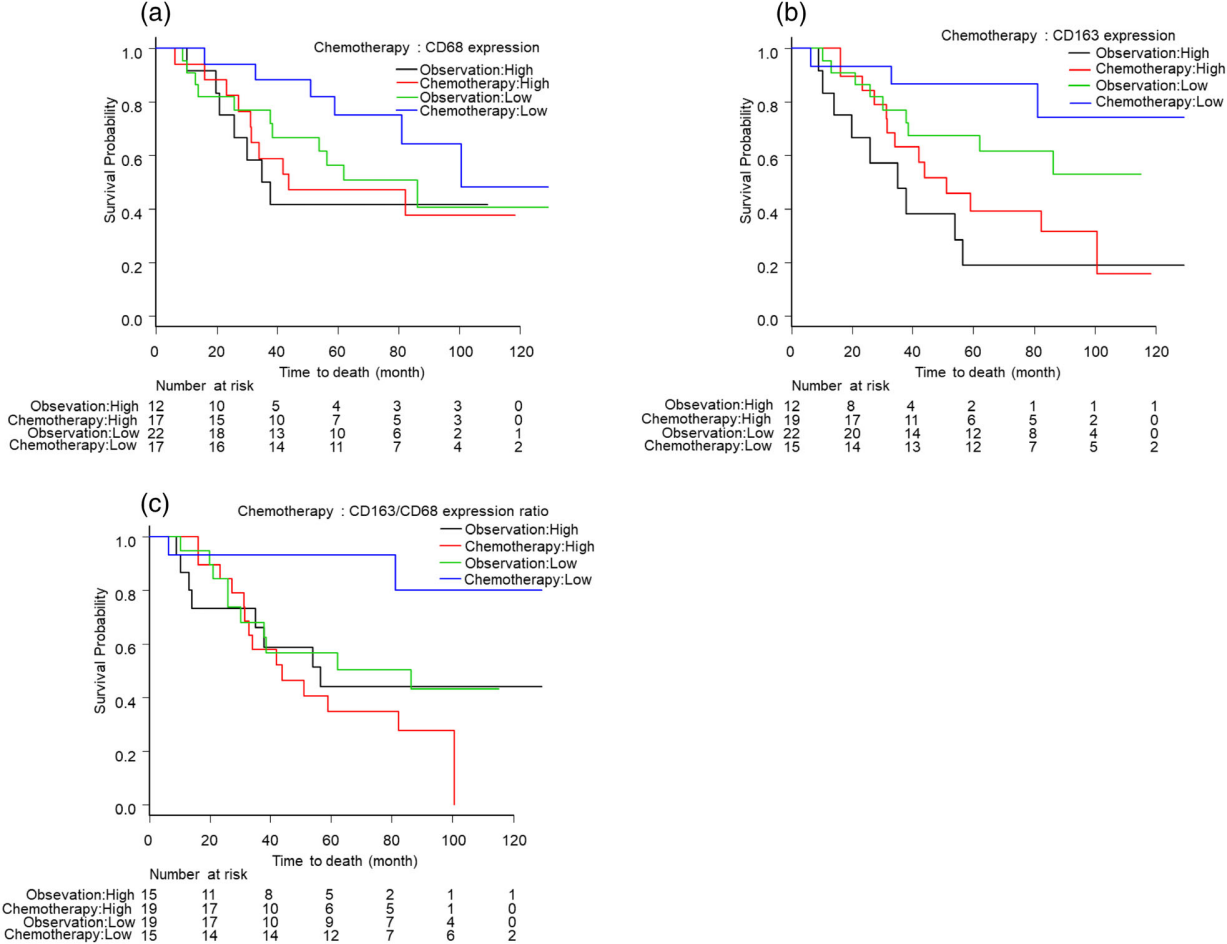
Overall survival Kaplan Meier curves based on the expression status of CD68 and CD163 and treatment. (a) Overall survival curves based on the expression status of CD68 and treatment. (b) Overall survival curves based on the expression status of CD163 and treatment. (c) Overall survival curves based on the expression ratio of CD163/CD68 and treatment.

According to PSM analysis, 34 pairs (*N* = 68, chemotherapy group: 34 patients, observation group: 34 patients) were created. The relationship between chemotherapy or observation with clinicopathological characteristics and the expression status of CD68 and CD163 before and after matching are shown in Table 5. Survival outcome after matching is shown in Table 6 and Figure 2. Patients with a low CD163/CD68 expression ratio significantly benefited from adjuvant chemotherapy (CD163/CD68 ratio: HR = 0.21, 95% CI: 0.05–0.96, *p* = 0.043). In contrast, patients with a high CD163/CD68 expression ratio obtained no survival benefit from adjuvant chemotherapy (CD163/CD68 ratio: HR = 1.34, 95% CI: 0.59–3.2, *p* = 0.515).

**TABLE 5:** The correlation between chemotherapy or observation with clinicopathological characteristics and the expression status of CD68 and CD163 before and after matching.

Variables	Before matching			After matching		
	Observation (*n* = 42)	Chemotherapy (*n* = 62)	Standardized mean difference	Observation (*n* = 34)	Chemotherapy (*n* = 34)	Standardized mean difference
Sex						
Female (7)	3 (42.9)	4 (57.1)	0.027	2 (50)	2 (50)	<0.001
Male (107)	39 (40.2)	58 (59.8)		32 (50)	32 (50)	
Age (average)	72.6	70.2	0.304	71.6	72.8	0.174
Smoking						
No (2)	1 (50)	1 (50)	0.055	1 (50)	1 (50)	<0.001
Yes (102)	41 (40.2)	61 (59.8)		33 (50)	33 (50)	
Stage						
I (57)	31 (54.4)	26 (45.6)	0.682	23 (48.9)	24 (51.1)	0.064
II/III (47)	11 (23.4)	36 (76.6)		11 (52.3)	10 (47.7)	
Pleural invasion						
No (76)	35 (46.1)	41 (53.9)	0.404	27 (50.9)	26 (49.1)	0.071
Yes (28)	7 (25)	21 (75)		7 (46.7)	8 (53.3)	
Lymphocytic invasion						
No (92)	38 (41.3)	54 (58.7)	0.107	30 (49.2)	31 (50.8)	0.097
Yes (12)	4 (44.4)	8 (66.7)		4 (57.1)	3 (42.0)	
Vascular invasion						
No (70)	33 (47.1)	37 (52.9)	0.418	25 (52.1)	23 (47.9)	0.129
Yes (34)	9 (26.5)	25 (73.5)		9 (45)	11 (55)	

**TABLE 6:** Survival outcome in months according to the expression status of CD68 and CD163 and treatment.

(A)			Overall survival			
Marker	No. of patients	Median (95% CI)	5 year OS (95% CI)	HR	95% CI	*p*‐value
Low CD68 expression	39					
Observation	22	86.2 (37.7‐NA)	56.5 (32.8–74.6)	1		
Chemotherapy	17	100.7 (59‐NA)	75.1 (43.6–89.9)	0.48	0.18–1.29	0.148
High CD68 expression	29					
Observation	12	36.3 (19.9‐NA)	41.7 (15.2–66.5)	1		
Chemotherapy	17	43.7 (27.4‐NA)	47.1 (23–68)	0.88	0.34–2.32	0.8

(B)			Overall survival			
Marker	No. of patients	Median (95%CI)	5 year‐OS (95%CI)	HR	95% CI	*p* value
Low CD163 expression	37					
Observation	22	NA (37.7‐NA)	67.4 (43.4–83)	1		
Chemotherapy	15	NA (81‐NA)	86.7 (56.4–96.5)	0.42	0.11–1.56	0.196
High CD163 expression	31					
Observation	12	34.9 (10.4–56.3)	19 (3–45.6)	1		
Chemotherapy	19	51.1 (31.3–100.7)	39.4 (17.5–60.7)	0.63	0.27–1.49	0.298

(C)			Overall survival			
Marker	No. of patients	Median (95% CI)	5 year OS (95% CI)	HR	95% CI	*p*‐value
Low CD163/CD68 expression ratio	34					
Observation	19	86.2 (25.9‐NA)	56.7 (31.6–75.6)	1		
Chemotherapy	15	NA (81‐NA)	93.3 (61.3–99)	0.21	0.05–0.96	**0.043**
High CD163/CD68 expression ratio	34					
Observation	15	56.3 (13.1‐NA)	44 (18.5–67.1)	1		
Chemotherapy	19	43.7 (31.3–82.3)	34.7 (14.5–56)	1.34	0.59–3.2	0.515

*Note*: Bold value indicates statistically significant *p* < 0.05.

Abbreviations: CI, confidence interval; HR, hazard ratio; NA, not available; OS, overall survival.

## DISCUSSION

TAMs are one of the key components in the TME, and M2 TAMs promote angiogenesis, wound healing, and tumor growth.^12^, ^19^ Increased numbers of M2 TAMs have previously been associated with a poor prognosis in breast cancer,^20^ bladder cancer,^21^ and classic cervical cancer.^22^ In our univariate analysis, high CD163 expression and a high CD163/CD68 expression ratio were both found to be significant prognostic factors for OS and predictive factors for recurrence. Multivariate analyses also revealed that CD163 expression and the CD163/CD68 expression ratio were independent prognostic factors for OS and predictive factors for recurrence. In previous studies, Lin et al. reported that positive CD163 expression was significantly correlated with poor disease‐free survival (DFS) and poor OS in stage I lung SCC.^3^ Sumitomo et al. described that the DFS and OS rates were significantly lower in patients with stromal M2 TAM‐high tumors and those with alveolar M2 TAM‐high tumors in NSCLC.^7^ In their study they found that the c‐reactive protein (CRP) level was associated with stromal and alveolar M2 TAM density levels.^7^ A previous clinical study also reported that a higher density of CD163‐positive macrophages was associated with elevated CRP levels.^23^ These results suggest the existence of crosstalk between cancer‐related inflammation and M2 TAMs in the TME.^8^, ^24^ During tumor progression, this crosstalk may produce more aggressive tumors. Previous clinical studies also reported that an elevated CRP level was a predictor of a worse prognosis in patients with NSCLC.^25^ In their study, Hwang et al. reported that TAMs were significantly associated with angiogenesis and lymphangiogenesis, contributing to the progression of NSCLC.^26^ They described that M2 macrophages significantly enhanced the protein and mRNA expression of vascular endothelial growth factor (VEGF)‐A and VEGF‐C.^26^ M2 TAMs may have multiple functions that induce tumor progression. Therefore, M2 TAMs may become a target for therapy in the future.

In addition, we found that patients with a low CD163/CD68 expression ratio within the tumor significantly benefited from adjuvant chemotherapy compared to patients with a high CD163/CD68 expression ratio within the tumor. Lan et al. also assessed the CD163/CD68 expression ratio and reported that platinum‐resistant recurrences were more prevalent in the high‐CD163/CD68 ratio group than in the low‐ratio group (*p* = 0.020), which suggested that activation of macrophages towards the M2 phenotype might correlate with types of recurrent disease in ovarian cancer (platinum‐resistant recurrence vs. platinum‐sensitive).^11^ Petrillo et al. found that the percentage of women showing pR0 after concomitant chemoradiation (CT/RT) was almost double in patients with high M1/M2 ratios compared to cases with a low M1/M2 ratio, and the ratio between M1 and M2 macrophages represented an independent predictor of pR0.^22^ They also reported that the differentiation of TAMs toward an M2 phenotype could promote the development of resistance to CT/RT in locally advanced cervical cancer patients.^22^ Dijkgraaf et al. reported that a chemotherapy‐mediated increase in tumor‐promoting M2 macrophages might form an indirect mechanism for chemoresistance, such as resistance to therapy. The increase in M2 macrophages was associated with increased levels of interleukin (IL)‐6 and prostaglandin E2 (PGE2), two inflammatory mediators known to skew the differentiation of monocytes to tumor‐promoting M2 macrophages.^27^ Recently, Kawaguchi et al. reported that treatment with nimesulide depleted M2‐like TAMs in the TME and enhanced the tumor inhibitory effects of cisplatin in a lung cancer model.^28^ Our results and previous studies suggest that M2 TAMs may be a useful predictive marker for the efficacy of adjuvant chemotherapy, although further studies are needed.

In the present study, high CD163 expression was frequently found in patients with PD‐L1 positive tumors (TC) (*p* = 0.029). Sumitomo et al. found that PD‐L1 expression on TCs and ICs was significantly higher in a stromal M2 TAM‐high group than in a stromal M2 TAM‐low group.^29^ It is well established that TAMs are immunosuppressive cells that induce drug resistance to PD‐1/PD‐L1 therapy. Several studies have revealed that TAMs contribute to T cell dysfunction and exhaustion through the secretion of cytokines and metabolic products,^30^, ^31^, ^32^ and by increasing PD‐L1 expression in tumor cells and other immunosuppressive cells.^33^, ^34^, ^35^ Therefore, immune checkpoint inhibitors and TAM‐targeting therapy will be required in the future to improve patient outcomes.

This study had some limitations. First, we used TMAs rather than large tissue sections. Although we checked immune cell infiltration before constructing the TMA, heterogeneous expression of CD163 and CD68 may have impacted our immunohistochemical results. Second, this was a retrospective study performed at a single institution; thus, the possibility of bias cannot be excluded. In addition, a second cohort should be used for validation analyses, and the population size evaluated in this study was small. However, decreases in cigarette smoking in developed countries may result in fewer cases of lung SCC.

In conclusion, our results showed that high CD163 expression and a high CD163/CD68 expression ratio were both significant prognostic factors for OS and predictive factors for recurrence. Furthermore, patients with a low CD163/CD68 expression ratio benefited more from adjuvant chemotherapy than patients with a high CD163/CD68 expression ratio. Thus, we suggest M2 TAMs may be a useful marker for predicting prognosis and differential benefit from adjuvant chemotherapy in patients with surgically resected lung SCCs.

## AUTHOR CONTRIBUTIONS

All authors had full access to the data in the study and take responsibility for the integrity of the data and the accuracy of the data analysis. Conceptualization: Naoki Yanagawa and Tamotsu Sugai. Methodology: Naoki Yanagawa and Tamotsu Sugai. Investigation: Naoki Yanagawa, Shunsuke Shikanai, Mayu Sugai, Yoshihiko Koike, Yoshinari Asai, Takayuki Tanji, Hajime Saito, and Makoto Maemondo. Formal Analysis: Naoki Yanagawa, Ryo Sugimoto, Mitsumasa Osakabe, and Noriyuki Uesugi. Resources: Tamotsu Sugai. Writing–original draft: Naoki Yanagawa. Writing–review and editing: Naoki Yanagawa and Tamotsu Suga. Visualization: Mitsumasa Osakabe. Supervision: Tamotsu Sugai.

## CONFLICT OF INTEREST STATEMENT

The authors declare that they have no conflicts of interest.

## CONSENT FOR PUBLICATION

We guarantee that (a) the work is original; (b) the work has not been and will not be published in whole, or in part, in any other journal; and (c) all of the authors have agreed to the contents of the manuscript in its submitted form.

## Data Availability

The data supporting the findings of the study are available from the corresponding author upon reasonable request.
